# A comparative study of two robotic thyroidectomy procedures: transoral vestibular versus bilateral axillary-breast approach

**DOI:** 10.1186/s12893-022-01609-9

**Published:** 2022-05-11

**Authors:** Qingqing He, Jian Zhu, Xiaolei Li, Meng Wang, Gang Wang, Peng Zhou, Dan Wang, Changrui Liu, Luming Zheng, Dayong Zhuang, Ziyi Fan, Fang Yu, Yunhan Ma, Xianjiao Cao, Suai Wang, Tao Yue, Jinzhi Hu

**Affiliations:** Department of Thyroid and Breast Surgery, The 960th Hospital of the PLA Joint Logistics Support Force (Former Jinan Military General Hospital of People’s Liberation Army), Jinan, China

**Keywords:** Papillary thyroid carcinoma, Transoral-vestibular robotic thyroidectomy, Bilateral axillo-breast approach robotic thyroidectomy, Remote-access thyroid surgery

## Abstract

**Objective:**

To compare the surgical outcomes between the transoral-vestibular robotic thyroidectomy (TOVRT) and bilateral axillo-breast approach robotic thyroidectomy (BABART).

**Methods:**

A total of 99 patients with papillary thyroid carcinoma but no distant metastasis were enrolled in this study from May 2020 to April 2021. Lobectomy or total thyroidectomy with central lymph node dissection were performed in all cases. All 99 patients were received an ultrasound guided fine needle aspiration biopsy prior to surgical intervention, out of which 49 patients underwent TOVRT, while rest 50 patients underwent BABART. During the procedure, intraoperative neuromonitoring system was used and all recurrent laryngeal nerves (RLNs) were preserved, additionally for TOVRT procedure, three intraoral ports or right axillary fold incision was used to allow for fine countertraction of tissue for radical oncological dissection. The clinical data including age, gender, height, weight, BMI, primary tumor size, number of central lymph node removed, central lymph node metastasis, operating time, total hospital stays, postoperative hospital stays, total postoperative drainage volume, postoperative pain score, cosmetic effect and complications were recorded and analyzed.

**Results:**

There were no significant differences in gender, height, weight, BMI and removed central lymph nodes between the two groups (*P* > 0.05). Patients accepted TOVRT were younger and had smaller primary tumor size than those who accepted BABART. The TOVRT group had a longer surgical time than the BABART group, but with smaller postoperative drainage volume and superior cosmetic effect (under visual analogue scale, VAS) (*P* < 0.05). There was no significant difference in lymph node metastasis, hospital stay and postoperative pain score (under numerical rating scale, NRS) between the two groups (*P* > 0.05). Last but not least, certain peculiar complications were observed in TOVRT group: paresthesia of the lower lip and the chin (one case), surgical site infection (one case) and skin burn (one case).

**Conclusion:**

Transoral-vestibular robotic thyroidectomy is safe and feasible for certain patients, which could be considered an alternative approach for patients who require no scarring on their neck.

## Introduction

Thyroid tumors are very common in young and middle-aged women [1]. In need of social contact, friendship, marriage, job promotion, privacy protection and such, a good cosmetic effect of postoperative surgical site on the neck has been desire by the patients. Robotic thyroid surgery, with the advantages of “cosmetic” and “minimally invasive”, has been prevailed in these days. Among various extra-cervical approaches for robotic thyroidectomy, the transoral-vestibular approach as a kind of “natural orifice transluminal endoscopic surgery (NOTES)” is drawing more attention and performed in increasing frequency lately due to less flap dissection and excellent cosmetic outcomes [2–7]. However, the safety and feasibility have not been concluded yet [8–10]. Recently, we performed several cases of robotic thyroid surgery via the transoral-vestibular approach using the Da Vinci Si Surgical System and would like to report our safety and feasibility study of transoral-vestibular robotic thyroidectomy (TOVRT).

## Patients and methods

### Patient eligibility and study design

From May 2020 to April 2021, the da Vinci Si surgical system (Intuitive Surgical, Inc., Sunnyvale, CA) was used to conduct robotic thyroidectomy at the 960th Hospital of the PLA Joint Logistics Support Force (Former Jinan Military General Hospital of PLA). The purpose of our study was to compare the surgical outcomes of the transoral-vestibular (49 cases) approach to conventional bilateral axillo-breast approach (50 cases).

### Inclusion criteria

Financial situation, patients’ preference, and in particular disease status affect the selection of surgical approach. Informed consent was obtained from each patient regarding the approach and research. We adhere to the “treatment first, function protection second, beauty third” principle in this study, and always avoid non-standard operation.

**Fig. 1:**
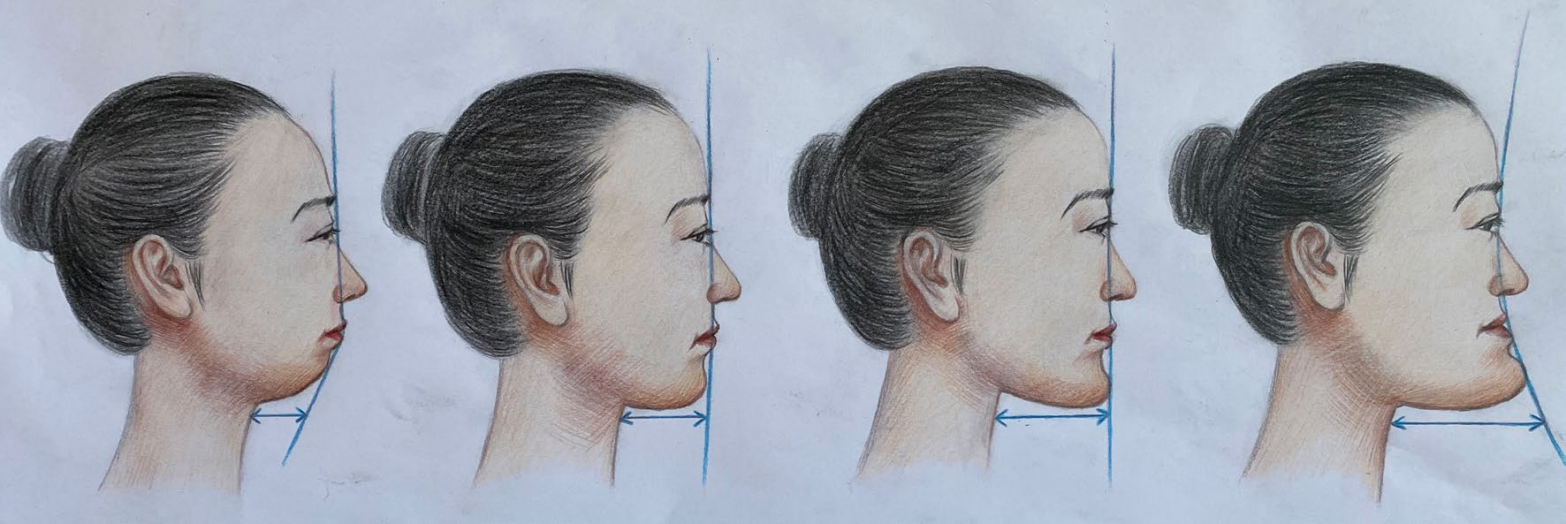
The schematic diagram of mandibular shape. Over prominent mandible was not suitable for transoral-vestibular robotic thyroidectomy

Institutional review committee at the 960th Hospital of the PLA Joint Logistics Support Force approved our proposal before the study started. According to the Chinese Expert Consensus for Robotic System Assisted Thyroid and Parathyroid Surgery and the Chinese Expert Consensus for Endoscopic Thyroid Surgery by Transoral-vestibular Approach, patients with thyroid tumor ≤ 2 cm can be considered for either TOVRT or BABA. Patients with a history of neck surgery, oral and/or neck infections, or overprominent mandible (Fig. 1) were excluded. Thyroid carcinoma with extrathyroidal extension (ETE), suspicious cervical lymph node metastasis in the lateral compartment, and distant metastasis were also among the exclusion criteria.

### Operative procedure of bilateral axillary-breast (BABA) approach

The BABA robotic thyroidectomy has previously been described in detail in the literature [11]. Parathyroid glands were transplanted in the sternocleidomastoid muscle or removed inadvertently once we found that the blood supply to the gland was compromised.

Four hours after surgery, the patient could be given liquid food. Routine mouth rinse should be adopted after meals. The drainage tube could be removed when the drainage volume was less than 10 ml over a 24 h period.

### Preoperative preparation of transoral-vestibular robotic thyroidectomy

Preoperative ultrasonography was used to evaluate thyroid volume outline, relationship between thyroid and adjacent organs, and lymph nodes in the central and lateral cervical regions. Vocal cord mobility should be evaluated with flexible laryngoscopy before surgery. One day before the surgical operation, patients’ mouth and teeth were cleaned and treated with strong tinidazole gargle for many times; 0.1 ml of carbon nanoparticles’ suspension injection (Chongqing Lummy Pharmaceuticals, China) was applied to the left and right leaf thyroids under the guidance of ultrasound, leaving the parathyroid glands unstained but staining the level VI area lymph nodes. 30 min before the surgical operation, prophylactic antibiotics (cephalosporins) were given intravenously. Patients who are allergic to penicillin or cephalosporins were treated with clindamycin to prevent infection.

### Operative procedure of transoral-vestibular robotic thyroidectomy [12, 13]

**Fig. 2:**
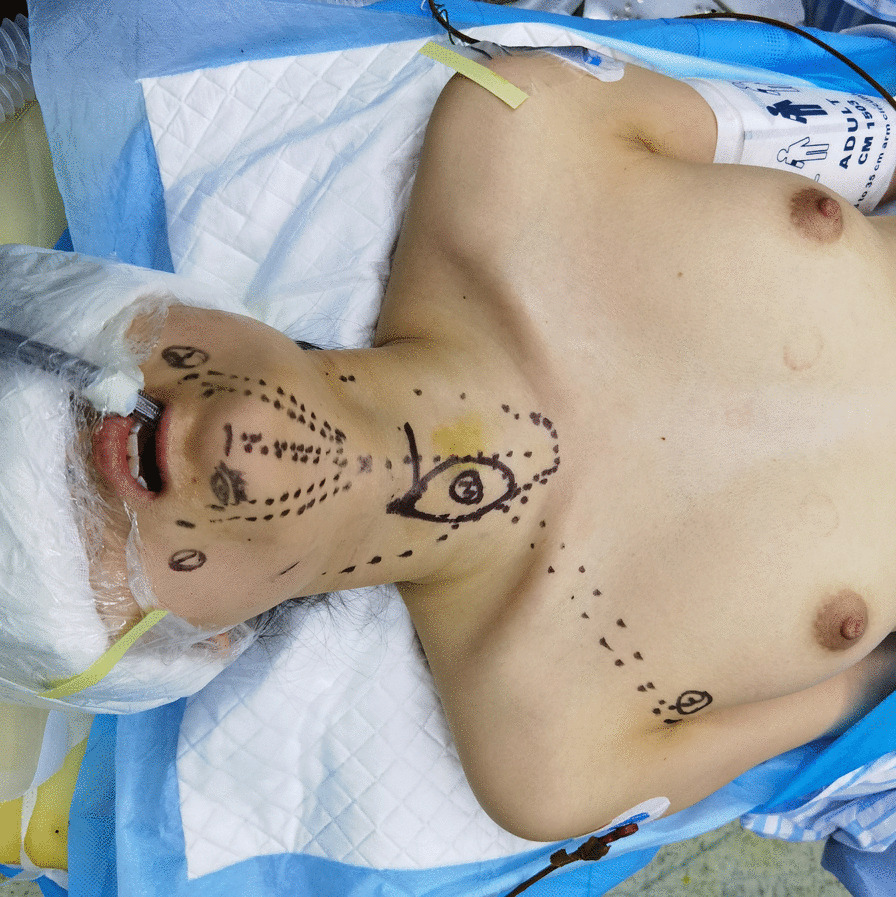
The patient’s position and auxiliary marker on the body surface. After general anesthesia, a supine position with neck extended was adopted, the eyes were covered with gauze and waterproof drape. The location of four trocars and corresponding pathway were marked on the body surface

**Fig. 3:**
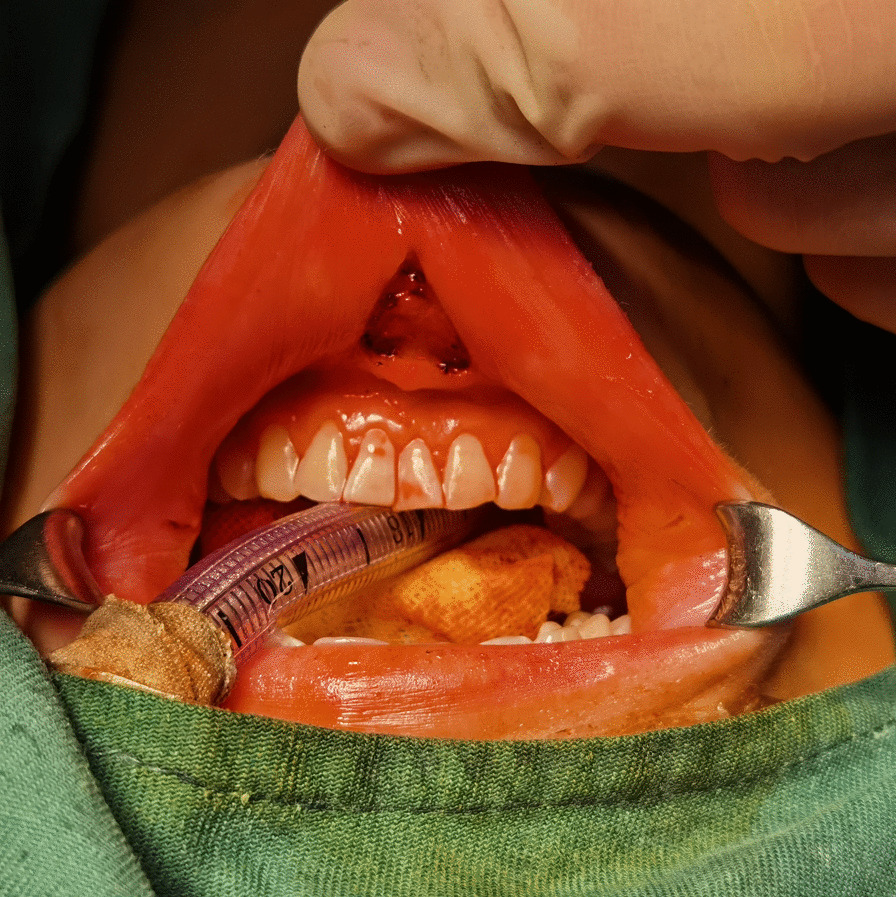
A “roof-shaped” transverse midline incision and two lateral oral vestibular incisions

The patient’ position and orotracheal intubation were shown in Fig. 2, with neck extended and lower limbs separated. A nerve monitoring tube (Shanghai NCC Medical Co., LTD, China) was used for general anesthesia through oral or nasal cannula. 0.1% retouch povidone iodine was adopted for oral cavity disinfection. With a 23-gauge spinal needle, we injected diluted epinephrine solutions (1:200,000, 10 ml) and ropivacaine hydrochloride (50 mg) into the subcutaneous areas for hydrodissection and less bleeding. A “roof-shaped” transverse midline incision at 1 cm above the frenulum labii inferioris, then two lateral incisions (5–8 mm) anterior to the lateral side of the fourth tooth or two lateral incisions through the mucosa at the corner of the mouth were made by a scalpel and monopolar electrocautery to prevent the mental nerve injury, followed by a blunt dissection using a vascular tunneler to the suprasternal fossa, creating a fan-shaped space in the submental area (Fig. 3). The first arm with dual-channel 30° face-down camera was placed through the “roof-shaped” transverse midline incision while the another two lateral effector arms were passed through the lateral oral vestibular incisions gently as the skin in the lower jaw is relatively fixed and thin.

**Fig. 4:**
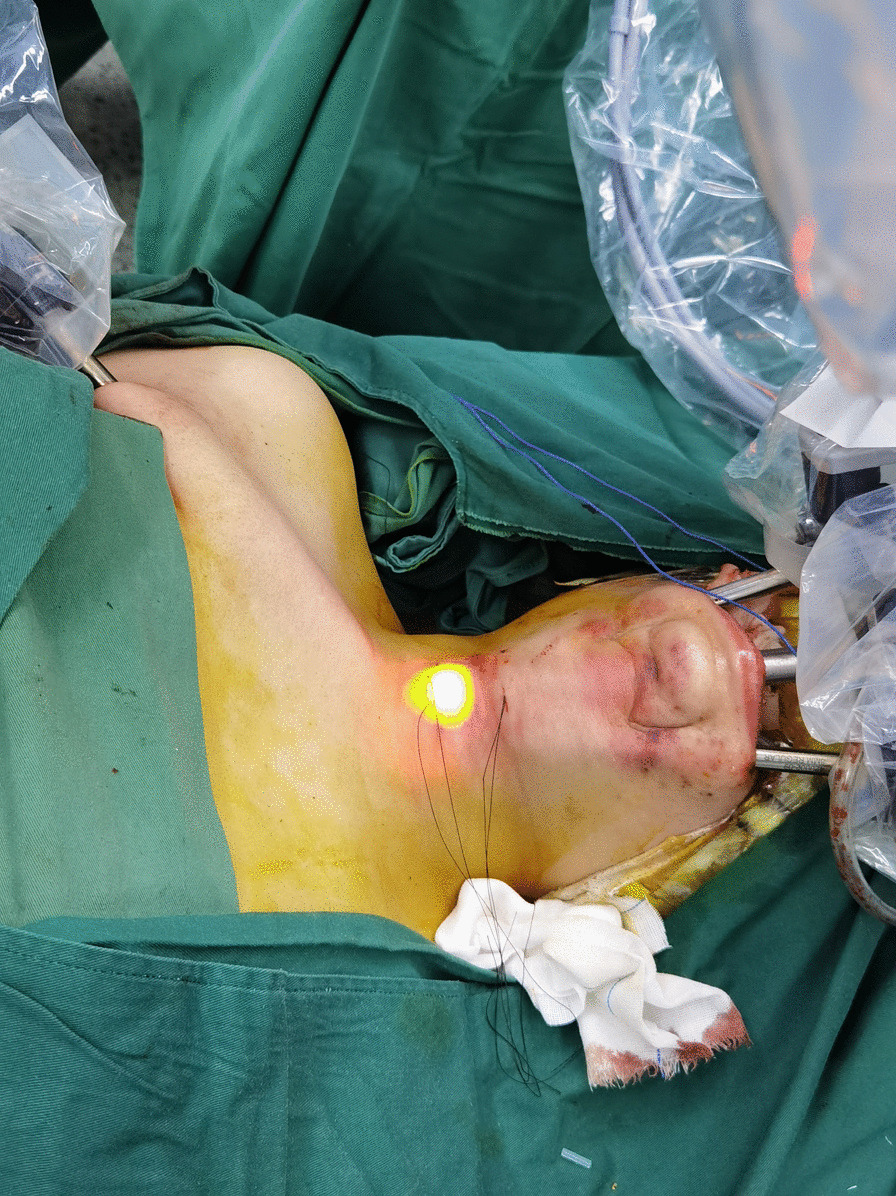
The docking of the da Vinci Si surgical system. Three trocars were inserted in the oral vestibule, and meanwhile the fourth trocar for was placed through the right axillary incision

How above three robotic arms were placed through the oral vestibular incision is shown in Fig. 4. A Prograsp or Maryland bipolar forceps was placed through the left port, and a harmonic curved shears was placed through the right port. To inflate and create the working space, 4–5 mmHg CO_2_ insufflation pressure was enough and the CO_2_ flow rate should be limited to 15 l/min or under. Under the view provided by the dual-channel 30° face-down camera, a working space was created using harmonic curved shears. The 5 mm Maryland dissector can be used for the right axillary fold incision based on the surgeon’s own discretion and thyroid size.

Elevate the skin flap by the harmonic curved shears and gradually extend to the suprasternal notch. The midportion of the sternocleidomastoid (SCM) muscle would be the outer boundary of the skin flap. With the help of Prograsp forceps and harmonic curved shears, the midline fascia of the strap muscles, the sternohyoid and sternothyroid muscles were dissected and the thyroid gland was exposed. Then we divided the middle thyroid vein and inferior vein, and dissected the pyramidal lobe and the isthmus from the trachea successively. When handing the superior thyroid vessels, superior laryngeal nerve and the superior parathyroid gland should be protected much carefully by dissecting the vessels close to the thyroid gland. The superior thyroid artery can be clipped by Hem-o-lock clips if necessary. Carbon nanoparticles injection is recommended into the thyroid glands for negative parathyroid imaging in order to identify and protect the superior parathyroid gland. The parathyroid gland should be protected by fine capsule anatomy techniques during the operation. Superior pole of thyroid gland was then retracted upward and medially, therefore the white and shiny recurrent laryngeal nerve can be easily identified using an intraoperative nerve monitor (IONM) as it enters the larynx with magnified 10× surgical view. The thyroid gland was lifted up with the Maryland forceps through the axillary port. The soft tissue around thyroid was dissected, but tracing the distal part of the recurrent laryngeal nerve with great care to preserve it. Proper application of the harmonic scalpel can successfully remove the thyroid gland from the trachea by dissecting the Berry's ligament. An endoplastic bag was used to collect the resected specimen from the operation area. For patients with large volume of glandular lobe, an additional axillary fold incision tunnel is also a good method to facilitate specimen removal, which could reduce the risk of specimen destruction or fragmentation. Subsequently, a therapeutic or prophylactic central compartment dissection was performed. During ipsilateral or bilateral central lymph node dissection, the central compartment lymph nodes were carefully dissected to avoid injuries to the recurrent laryngeal nerve and parathyroids. We dissected the level VI lymph nodes (delphian/prelaryngeal, pretracheal, and paratracheal lymph nodes) using the robotic system.

**Fig. 5:**
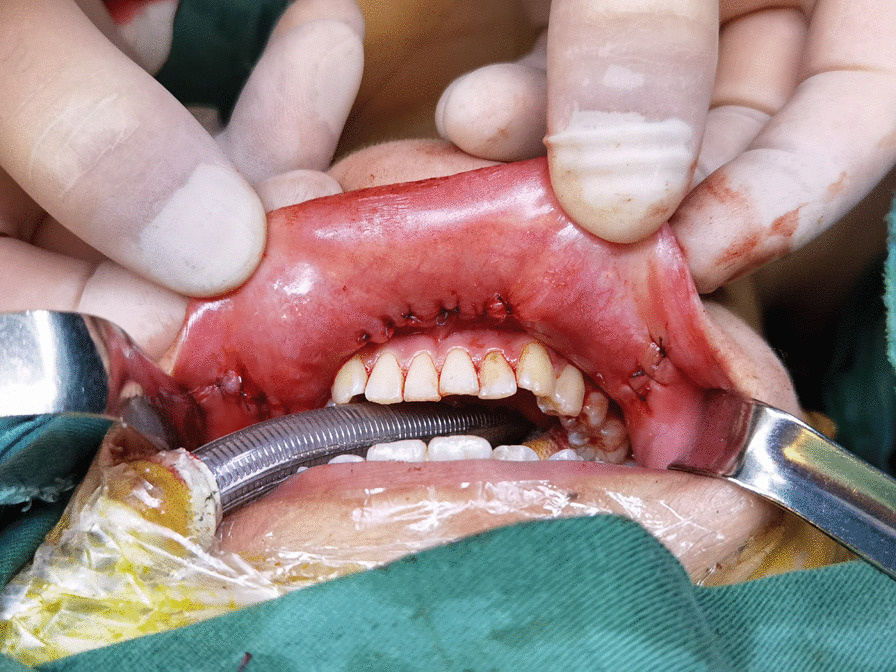
The oral vestibular mucosal incisions were sewed up with absorbable sutures

**Fig. 6:**
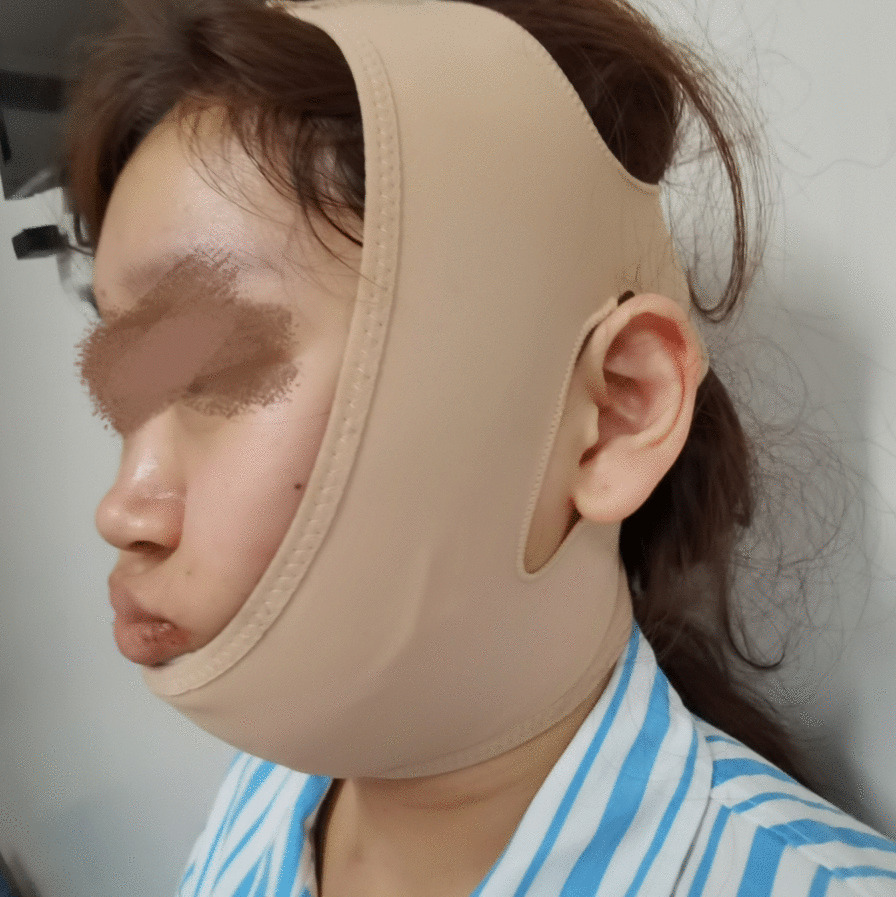
A pressurized headband was used to bandage the surgical area

According to the Chinese Guidelines for Diagnosis and Treatment of Thyroid Nodules and Differentiated Thyroid Cancer, prophylactic central node dissection is recommended under the condition that effective preservation of parathyroid gland and recurrent laryngeal nerve can be achieved. As a matter of fact, in our clinical practice we found the rate of central lymph node metastasis was nearly 50% and papillary thyroid microcarcinoma also had non-negligible metastasis rate. The lymph nodes behind the right recurrent laryngeal nerve should be removed in the case of right lobe thyroid carcinoma was also found. Application of carbon nanoparticles suspensions injection can stain the center compartment lymph nodes while leaving the parathyroid glands unstained, thus helping us identify and protect parathyroid glands and the recurrent laryngeal nerve, as well as facilitating the identification and clearance of lymph nodes. Special attention should be paid to ensure the heated harmonic scalpel stays 3 mm away from the recurrent laryngeal nerve and visible parathyroids. In transoral-vestibular robotic thyroidectomy, most surgeons use a left-handed instrument to pull the tissue and a right-handed instrument to perform the operation. However, the presence of thyroid cartilage complicates above techniques especially for beginners, whenever possible the left and right arms could be exchanged for ease of operation. Paraffin oil should be used to smear the lip during surgery to reduce the friction between the instrument and the lip mucosa which may result in oral mucosa injury. Added flexibility provided by the robotic serpentine multiarticular instruments allows for delicate operations in transoral-vestibular robotic thyroidectomy. Before sewing up the strap muscles using absorbable sutures, the surgical area and subcutaneous tunnel were douched by 3000 ml sterile distilled water (42 °C). Vacuum-assisted draining system should be placed in the operative area though the 3 mm small incision at the concealed lower margin of the mandible, through oral angle incision, or the axilla tunnel. Finally, we stitched incisions in oral vestibular mucosal (Fig. 5). After the operation, the surgical area was bandaged with a pressurized headband (Fig. 6).

### Statistical analysis

We evaluated patient demographic information such as age, gender, height, weight, BMI, as well as treatment information including primary tumor size, number of central lymph node removed, central lymph node metastasis, operating time, total hospital stays, postoperative hospital stays, total postoperative drainage volume, postoperative pain score, cosmetic effect and complications. The cosmetic effect was evaluated by Visual analogue scale (VAS) 1 month after the surgery. The pain was evaluated by numerical rating scale (NRS), with a score of 0–10 points where 0 is no pain and 10 is the worst pain imaginable. General clinical information of the patients is outlined in Table 1.

**Table 1:** The clinicopathological and surgical data between transoral and BABA robotic thyroidectomy groups

Items	TOVRT (n = 49)	BABART (*n* = 50)	Statistical values	*P*
**Age (years)**	35.0 ± 10.6	44.6 ± 11.8	0.000	< 0.05
Male/female	8/41	13/37	0.239	> 0.05
Height (cm)	163.4 ± 15.0	165.0 ± 8.0	0.473	> 0.05
Weight (kg)	63.6 ± 13.3	64.8 ± 11.5	0.468	> 0.05
BMI (kg/m^2^)	25.2 ± 14.2	23.7 ± 3.1	0.517	> 0.05
**Primary tumor size (mm)**	3.50 ± 3.3	5.56 ± 5.0	0.037	< 0.05
Number of removed central lymph nodes (bilateral)	(n = 7) 14.14 ± 2.968	(n = 16) 16.06 ± 6.981	1.851	> 0.05
Number of removed central lymph nodes (unilateral)	(n = 42) 7.85 ± 4.924	(n = 34) 8.26 ± 5.451	0.653	> 0.05
Central lymph node metastasis	0.51 ± 1.10	1.32 ± 2.6	0.082	> 0.05
**Operating time (min)**	(n = 17)	(n = 36)		
(Total thyroidectomy)	241.1 ± 77.8	153.4 ± 59.9	0.000	< 0.05
**Operating time (min)**	(n = 32)	(n = 14)		
(Lobectomy)	190.0 ± 44.4	121.0 ± 34.6	0.000	< 0.05
Total hospital stays (days)	9.53 ± 2.9	10.06 ± 3.1	0.317	> 0.05
Postoperative hospital stays (days)	7.4 ± 2.5	7.6 ± 2.1	0.249	> 0.05
**Total drainage volume (ml)**	153.1 ± 65.5	340.1 ± 140.2	0.000	< 0.05
NRS scores	2.47 ± 0.680	2.57 ± 0.791	0.456	> 0.05
**VAS scores**	8.79 ± 0.693	8.10 ± 0.848	0.000	< 0.05
Location of tumor			0.228	> 0.05

All statistical analysis was carried out with SPSS (SPSS 26.0). For continuous variables, if the data is normally distributed, Student’s *t* tests was used, otherwise, rank sum test was applied. For categorical variables, the chi-square test and Fisher’s exact test were used. Differences were considered statistically significant with *P* < 0.05.

## Results

All transoral vestibular robotic thyroidectomy and BABA robotic thyroidectomy were performed by the same two surgeons. The clinicopathologic characteristics of the patients in the transoral and BABA groups are shown in Table 1. Transoral vestibular robotic procedure was performed in 49 patients, and the BABA procedure was performed in 50 patients. There was no conversion from the robotic surgeries to open surgeries. Lobectomy or total thyroidectomy with central lymph node dissection was performed in all cases. During the procedure, intraoperative neuromonitoring system was used and all recurrent laryngeal nerves (RLNs) were preserved. Postoperative pathology confirmed that all patients had papillary thyroid carcinoma (PTC).

There differences in gender, height, weight, BMI, removed central lymph nodes, central lymph node metastasis, total hospital stay, postoperative hospital stay, NRS scores and location of tumor were not significant between the two groups (all *P* > 0.05). Patients accepted TOVRT were younger and had smaller primary tumor size than those who accepted BABART. The TOVRT group had a longer surgical time than the BABART group regardless of whether total thyroidectomy or lobectomy was conducted, but with smaller postoperative drainage volume and superior cosmetic effect (8.79 ± 0.69 vs. 8.10 ± 0.85, *P* < 0.05). It also should be noted that there were some peculiar complications such as paresthesia of the lower lip and the chin (one case), surgical site infection (one case) and skin burn (one case) in transoral thyroidectomy.

## Discussion

**Fig. 7:**
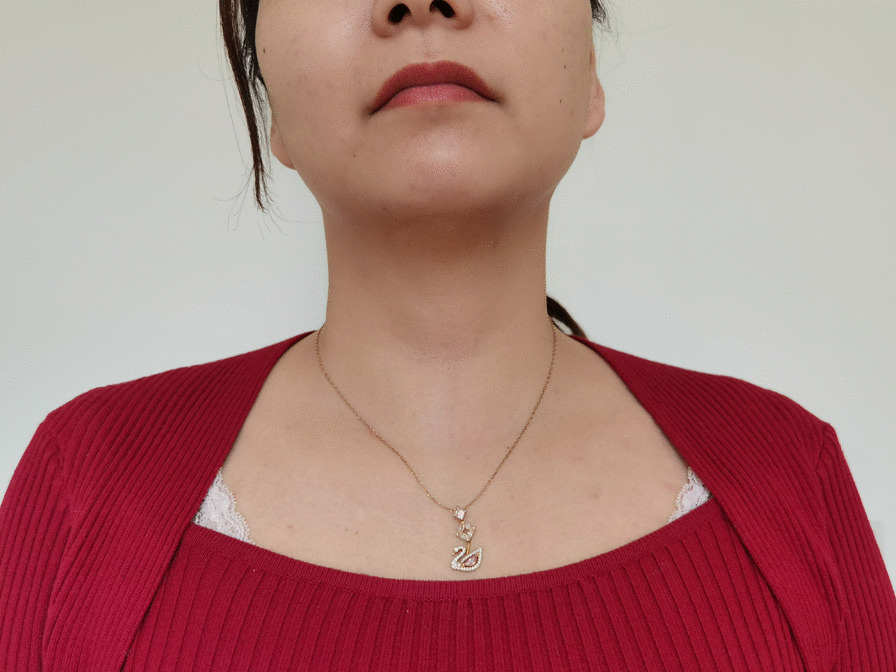
Three months after transoral-vestibular robotic thyroidectomy, a photograph was taken

Conventional open thyroidectomy is considered the most secure procedure in general surgery, despite the finding that an anterior cervical neck scar can negatively impact patient quality of life. Many extra-cervical approaches to the thyroid have been described to circumvent anterior neck scarring, including the trans-axillary, robotic-facelift, and transoral endoscopic vestibular approaches [12–15], but these incisions are not truly scar-free. Transoral thyroidectomy is a form of NOTES (natural orifice transluminal endoscopic surgery) [16] technique with minimal invasiveness and no scar on body surface (Fig. 7), including vestibular approach and sublingual access [17, 18], but the latter is obsolete because of the structural damage it may cause. The thyroid gland is a solid organ rich in blood, is adjacent to the complex anatomical structure and important blood vessels and nerves, and has no natural space around it. Hence to create working space without damaging the surrounding mental nerves there put forward a higher demand on the surgeons. The flap dissection is the most difficult part in the surgical procedure and proves to be technically challenging as a result of the imperceptible craniocaudal view and confined neck workspace. It’s common to dissect the sternocleidomastoid muscles to the upper layer with platysma in the early stage of our study. Therefore, a comprehensive knowledge of anatomical structures is desired and an external hanging central neck suture is routinely applied for better exposure. After the superior thyroid artery and vein ligated with ultrasonic energy device and the upper pole of thyroid lifted up, it’s very easy to identify the RLN with IONM, since the location of RLN insertion to the larynx is relatively fixed. Transoral-vestibular thyroidectomy has only been performed in South Korea, with a clinical study of 304 patients at most, and few in China, but the surgical outcome is uncertain [19]. From the experience of endoscopic surgery for the protection of recurrent laryngeal nerve and parathyroid gland, no serious complications occurred in transoral-vestibular robotic thyroid surgery. However, one common complication of transoral-vestibular thyroid surgery is numbness of the lower lip and chin skin due to mental nerve overstrain or injury, due to the fact that the trocar tunnel is close to the branch of the mental nerve. We made the incision of the operation aperture as close to the corner of the mouth as possible, as a result in our study only one patient had paresthesia of the lower lip and the chin, which effectively reduced the occurrence of complications. Another complication is postoperative infection. In our study, the original class I incision was changed to class II incision through oral approach. Preoperative prophylactic use of antibiotics and postoperative routine drainage can effectively prevent the occurrence of postoperative infection. Other robot-related complications, such as lacerated mouth injuries, facial contusions on the cheekbones, and perforated skin on the chin need to be noted as well. However, these complications are closely related to the operator’s experience and can generally be avoided with appropriate protection and careful procedures [20–22].

Robotic system benefits surgeons in many ways: the 3D HD magnified vision broke the limit of the naked eye, the endowrist surgical instruments exceeded the functional limit of human hands, the intuitive manipulation facilitates eye-hand coordination and the real-time hand-device tip synchronization allows surgeons to intuitively manipulate the device and helps surgeons fully utilize their open-procedure experience. Their mechanical arms have filtering, anti-shaking, anti-error, conversion functions, etc., features to operate freely in a narrow working space over human hand. Nevertheless, in transoral-vestibular thyroidectomy, the craniocaudal direction visual field is different from that of BABA robotic thyroidectomy or traditional open thyroidectomy. The surgeon to operate the robot needs to receive a rigorous and standardized training.

The additional axillary arm incision is mainly used to pull the band muscle and thyroid tissue to make the operation easier. An additional axillary fold incision tunnel is also a good method to facilitate specimen removal, which could reduce the risk of specimen destruction or fragmentation, and can be used for postoperative drainage tube placement. But the axillary fold incision left obvious skin scar on the body surface. For more difficult surgical cases, such as greater thyroid nodule and dorsal thyroid nodule near the recurrent laryngeal nerve, an additional axillary arm can help pull the thyroid and remove the resected specimen [23]. For the time being, if the additional axillary arm incision is used for the transoral-vestibular robotic thyroidectomy, indications are as follows [7, 24–26]: ① a strong need for thyroid surgery without scar on the neck; ② benign tumor ≤ 6 cm (single, multinodular goiter or follicular neoplasm); ③ thyroid papillary carcinoma ≤ 1 cm without lateral cervical lymph node metastasis; ④ concave face shape. The contraindications are as follows: cervical disease, cannot maintain the head-backward position; oral ulcers; contraindication of anesthesia; previous history of neck surgery or radiotherapy; obvious invasion of trachea, esophagus or recurrent laryngeal nerve in malignant tumors. This additional axillary arm approach may enlarge the indications of transoral-vestibular robotic thyroidectomy treatment while maintaining good cosmetic effect. The shorter dissection distance may reduce postoperative pain.

Even with the improvement of the technical level of the robotic systems, we must still adhere to the “treatment quality first, minimally invasiveness and cosmetic outcome second” principle. Aesthetics are of course an important consideration, but they should not be at the expense of surgical safety and radical treatment of the tumor. In this study, we evaluated the safety and efficacy of the transoral-vestibular robotic thyroidectomy, focusing on safety and complications. Complications such as temporary and permanent hypoparathyroidism, permanent vocal cord palsy, hematoma, and seroma were not present in both of our transoral-vestibular and BABA robotic thyroidectomy procedures.

The most common complications in thyroid surgery are hypocalcemia and recurrent laryngeal nerve injury. Robotic transoral-vestibular thyroidectomy allows surgeons to better identify and preserve the superior parathyroid gland in situ. The routine application of recurrent laryngeal nerve monitoring technology during the operation is very effective and useful for the surgeon to quickly identify and confirm the journey of recurrent laryngeal nerve and avoid injury in the narrow operating space. Few important points to ponder below. First, the surgical time of transoral-vestibular thyroidectomy is significantly longer than that of BABA robotic thyroidectomy, accompanied by prolonged anesthesia time and increased cost. However, with the accumulation of clinical experience, the operation time can be gradually shortened. The mean operative time of transoral-vestibular thyroidectomy ranged from 113 to 386 min, with an average of 205.5 ± 61.9 min. Secondly, robotic manipulators do not have strong feedback, which may increase the risk of oral tears. During the procedure, the assistant pays close attention to the tension in the mouth to remind the surgeon to avoid rough handling. Pull both sides of the mouth outwards and make a longitudinal incision of 8 mm at the mucous membrane of both corners of the mouth to avoid mental nerve injury. Thirdly, oral approach leaves the body surface absolutely no scar, but the wound changes from the type I incision to the type II incision, increasing the chance of infection. Fortunately, no serious postoperative infections occurred in our study. Finally, lateral cervical lymph node dissection is difficult in transoral-vestibular approach due to the proximity to the lateral cervical region. Therefore, it is very necessary to fully evaluate the cervical lymph node metastasis of thyroid cancer patients preoperatively. The concept of “do no harm” must be held in the early stages of transoral-vestibular robotic thyroidectomy exploration [14, 27–30].

The advances in robotics and superior surgical field vision robotic system provides have made it possible to perform thyroid surgery using a new extra-cervical surgical approach. Our experience shows that transoral-vestibular robotic thyroidectomy is safe and feasible for selected patients, and is likely to be the development trend of minimally invasive thyroidectomy, but oncological safety always takes precedence over the cosmetic result. Transoral-vestibular robotic thyroidectomy has the potential to improve the 140-year-old Kocher’s classic cervical approach, but like any new surgical approach, it requires comprehensive data analysis and has a steep learning curve. Robotic surgeons need to have strong visual thinking, anatomical visual control in the face of enlarged local anatomical operation area and human–machine integration capability. Last but not least, there are still some unanswered questions regarding robotic transoral-vestibular thyroidectomy, including the design and manufacture of specialized equipment, long-term follow-up results, and the inability to perform lateral neck dissection. Therefore, the advantages and disadvantages of this new technique need to be fully explored through comprehensive data analysis from prospective, randomized, controlled, multi-institution studies.

## Conclusion

Transoral-vestibular robotic thyroidectomy is a safe and effective surgical procedure for patients who require no scarring on their neck.

## Data Availability

We do not wish to share our data supporting our findings because our hospital belongs to the PLA Joint Logistics Support Force, and we have special management regulations to restrict datasets disclosure.

## Abbreviations

TOVRT: Transoral-vestibular robotic thyroidectomy
BABART: Bilateral axillo-breast approach robotic thyroidectomy
PTC: Papillary thyroid carcinoma
VAS: Visual analogue scale
NRS: Numerical rating scale
SCM: Sternocleidomastoid
CND: Central neck dissection
IONM: Intraoperative nerve monitor
RLN: Recurrent laryngeal nerve
